# The quality of COVID-19 systematic reviews during the coronavirus 2019 pandemic: an exploratory comparison

**DOI:** 10.1186/s13643-024-02552-x

**Published:** 2024-05-08

**Authors:** Kevin T. McDermott, Mark Perry, Willemijn Linden, Rachel Croft, Robert Wolff, Jos Kleijnen

**Affiliations:** 1 Kleijnen Systematic Reviews Ltd, York, UK; 2 KSR Evidence Ltd, York, UK

**Keywords:** Systematic reviews, Evidence review, Quality appraisal, COVID-19, ROBIS, Methodology

## Abstract

**Background:**

The unprecedented volume and speed at which COVID-19-related systematic reviews (SRs) may have been produced has raised questions regarding the quality of this evidence. It is feasible that pandemic-related factors may have led to an impairment in quality (reduced internal validity, increased risk of bias [RoB]). This may have serious implications for decision-making related to public health and individual healthcare.

**Objective:**

The primary objective was to compare the quality of SRs published during the pandemic that were related to COVID-19 with SRs published during the pandemic that were unrelated to COVID-19 (all of which were fully appraised in the KSR Evidence database of SRs in healthcare). Our secondary objective was to compare the quality of SRs published during the pandemic (regardless of research topic), with SRs published pre-pandemic.

**Methods:**

We compared all SRs related to COVID-19 to all SRs unrelated to COVID-19 that (i) were published during the pandemic (between 1st March 2020 and September 14, 2022), (ii) were included in KSR Evidence, and (iii) had been appraised using the ROBIS tool. We then compared all SRs published during the pandemic (regardless of research topic) with a pre-pandemic sample of SRs.

**Results:**

For SRs published during the pandemic, we found there was no statistically significant difference in quality between those SRs tagged as being related to COVID-19 and those that were not [relative risk (RR) of low RoB for COVID-19 versus COVID-19-unrelated reviews: 0.94; 95% confidence interval (CI): 0.66 to 1.34]. Generally, COVID-19 SRs and COVID-19-unrelated SRs were both of low quality with only 10% of COVID-19 reviews and 11% of COVID-19-unrelated reviews rated as low RoB. However, SRs (regardless of topic) published during the pandemic were of lower quality than those published pre-pandemic (RR for low RoB for ‘during pandemic’ versus ‘pre-pandemic’: 0.30; 95% *CI*: 0.26 to 0.34) with 11% of pandemic and 36% of pre-pandemic SRs rated as low RoB.

**Conclusion:**

These results suggest COVID-19 and COVID-19-unrelated SRs published during the pandemic are equally of low quality. SRs published during the pandemic were generally lower quality compared with SRs published pre-pandemic irrespective of COVID-19 focus. Moreover, SR quality in general is seriously lacking, and considerable efforts need to be made to substantially improve the quality and rigour of the SR process.

## Background

The novel coronavirus, first identified in Wuhan province, China, in December 2019, and later named the severe acute respiratory syndrome coronavirus 2 (SARS-CoV-2, causing the disease described as COVID-19), led to dramatic worldwide impact. The World Health Organization (WHO) declared the outbreak to be a public health emergency of international concern on 30 January 2020 [1], and a worldwide pandemic was declared on 11 March 2020 [2]. This resulted in an unprecedented level of measures being introduced across the globe to curb the spread and impact of the disease. Estimates initially suggested the fatality rate could be as high as 15% in certain populations; however, as of March 2023, the case fatality rate ranges from 0.1 to 4.9% across the 20 countries most affected by COVID-19 [3]. The WHO declared the coronavirus emergency officially over on 05 May 2023 while emphasising that it remains a global threat.

The nature of the COVID-19 pandemic has led to a large volume of research at an accelerated pace, and this research has been the basis for a multitude of decisions regarding healthcare, as well as for numerous governmental policies to slow the spread of the pandemic and mitigate economic, social, and public health damage. However, the speed and volume of such a publication output have raised concerns that the body of evidence may become diluted with lower quality research [4], and given the impact of such research as a tool to inform policy on diverse areas such as healthcare, economics, and social behaviour, this is an issue of note. Evidence-based medicine, and indeed evidence-based decision-making in general, is an essential component of a rational, logic-driven society. However, despite systematic reviews (SRs) in science, medicine and healthcare being considered the top of the evidence pyramid and the highest calibre of evidence, the consistency and quality of reviews may be limited, with many published SRs being of lower quality, leading to high risks of bias or wasted resources [5, 6]. Additionally, ‘umbrella reviews’ (variously titled but may be described as systematic reviews of systematic reviews, overviews or reviews, summary of reviews, synthesis of reviews) are emerging as an important new methodology in the evidence synthesis toolkit. This is largely because of the increasing number and complexity of SR’s/MA that are available in the literature and a need for systematic consolidation of this evidence. The purpose of the ‘umbrella review’ is to identify, summarise, and, where appropriate, to analyse the evidence available in existing relevant SR’s/MA’s. It aims to provide a rigorous and systematic insight into this existing evidence, to highlight strengths and limitations, to identify contradictions and consistencies, and to generate an overall interpretation based on the range of included evidence [7]. Umbrella reviews are gaining prominence and are increasing in number each year [8]. As a consequence, the importance of SRs is again emphasised with a need for consistent high standards in execution and reporting of SR’s.

High-quality SR research relies upon not only the correct methodological design and execution but also, crucially, the clear reporting of these processes. Internal validity, external validity, and reporting quality are the three components which determine the production of overall ‘quality’ and the reliability, accuracy, and relevance of a SR. Briefly, internal validity depends on the machinery of the research — the study design, implementation, execution, and analysis. External validity relates to application and relevance and whether the research design can answer the research question. Finally, reporting quality refers to the clear description and explanations of the research, its hypotheses, design, execution, analysis, and interpretation. This relates to ensuring that the data and findings can be disseminated with clarity, relevance, and confidence. These components fundamentally regulate the notion of ‘quality’, and the impact of quality at the most explicit level, being concerned with the question of ‘to what extent can SR data be used and trusted to answer a particular question [9].’

Methodological quality of SRs can be assessed by examining the RoB, while reporting quality can be examined by adherence to reporting guidelines. The Preferred Reporting Items for Systematic Reviews and Meta-Analyses (PRISMA) checklist contains items which aim to ensure that a minimum standard of detail, reporting, and clarity is provided in the reporting of a SR [10]. The Cochrane handbook provides guidance and detail on the correct design and reporting of SRs [11], and multiple critical appraisal tools exist to assess methodological quality and determine the RoB [12]. The pressing need for rapid evidence generation at the SR level to inform decisions around COVID-19 may have led to impaired quality with higher likelihood of error and bias.

In accordance to this, studies have found that the quality of COVID-19-related research studies (including but not limited to SRs) is inferior to matched control studies that are not COVID-19 related [13, 14], and specific attempts to explore the quality of COVID-19 reviews have been published by several research teams [15–18]. An overview of SRs found that only three from a sample of 280 COVID-19-related SRs were of moderate or high quality according to AMSTAR 2 [19]. However, these studies did not compare the quality of COVID-19-related reviews with COVID-19-unrelated reviews over the same period, nor did this compare pandemic reviews with pre-pandemic reviews, or use reviews from beyond the earliest stages of the pandemic.

Given that roughly half of clinical practice guidelines are informed by SRs [20], it is important to understand the extent to which COVID-19-related SRs and SRs published during the pandemic more generally suffer from methodological problems relative to SRs on other topics or published pre-pandemic, respectively.

### Objectives

The main objective was to conduct an exploratory comparison of the quality of SRs related to COVID-19 with the quality of SRs not related to COVID-19, both of which were published during the same timeframe of the pandemic. The second objective was to compare the quality of SRs published during the pandemic (regardless of topic) with those published before the pandemic. We had two principal questions related to the objective which this work aimed to address:

1. How does overall quality of COVID-19-related SRs compare to those of COVID-19-unrelated SRs, both of which were published during the pandemic?
2. How does overall quality of SRs published during the pandemic (regardless of topic) compare to those SRs published prior to the pandemic?

Additionally, we also asked the following post hoc questions:

3) How does the review type distribution of COVID-19-related SRs compare to those of COVID-19-unrelated SRs published during the pandemic?
4) How does the review type distribution of SRs published during the pandemic compare to those SRs published prior to the pandemic?

## Methods

### Evidence identification

To conduct this exploration, we obtained two datasets from KSR Evidence. KSR Evidence is a database of SRs, meta-analyses, and health technology assessment reports published worldwide in healthcare since 2015, dating back further to 2010 for a selected group of topics. Approximately, 11% of the total number of SRs in the KSR Evidence database have so far been critically appraised using ROBIS [21]. SRs can be accelerated for appraisal at the request of KSR Evidence subscribers who may wish to use the SR in their research.

At the time of writing (October 2023), there are 262,278 SRs contained within the KSR Evidence database. Of these, 29,500 have been formally appraised. Both the numbers of database records, and the number of formally appraised SRs, are continuously increased. When SRs are identified and imported into KSR Evidence, they are screened according to priority. This consists of a process whereby records are initially examined and determined to warrant fast-track appraisal or not. Typically, factors such as journal prestige, subject area, and authorship reputation determine whether they are marked as ‘priority screen’. SRs not marked as priority enter the regular stream for appraisal in normal time and are not fast tracked unless specifically requested by a subscriber. Both datasets consisted of SRs that had been initially marked as ‘priority screens’.

ROBIS is a tool for evaluation of the RoB of SRs. ROBIS measures quality in four domains of systematic reviewing methodology: the criteria for eligibility of included studies, the methodology used to identify and/or select studies, the methodology used to collect and appraise studies, and the methodology used for the synthesis and interpretation of data. Each domain contains 5–6 signalling questions which interrogate the domain-specific methodology. ROBIS, which was developed by researchers of the University of Bristol collaborating with several institutions, including Kleijnen Systematic Reviews, is acknowledged as a valid and reliable tool for this purpose [22]. Records on KSR Evidence provide data on the RoB of each ROBIS domain, as well as the overall RoB. These are graded as ‘low, ‘high’, or ‘unclear’. Typically, where any item is appraised as ‘unclear’ or ‘high’ RoB, the entire domain is then appraised as such. Where any domain is appraised as ‘unclear’ or ‘high’, the overall quality of the SR is generally appraised to be as such. The overall RoB is the datapoint used in this study, and while we acknowledge the insights that could potentially be gained from considering the appraisals of each item within each domain, this is beyond the scope of this initial exploratory analysis. However, additional investigation will constitute an update to this research and will be reported in the future.

### Selection and screening

Dataset 1 consisted of all appraised SRs related to COVID-19 that had been uploaded to KSR Evidence during the most prominent period of the COVID-19 pandemic: 01 March 2020 to 14 September 2022. 14th September 2022 was the date that it was announced by the WHO that the end of the pandemic was in sight and therefore was chosen for this reason [23]. Dataset 1 also consisted of appraised SRs that had been uploaded to KSR Evidence during the same time period that were unrelated to COVID-19. All uploaded SRs where either published articles or available as advance E-publications within this date range.

Dataset 2 consisted of all appraised SRs on any topic that had been uploaded to KSR Evidence during 2018. That year was chosen to provide insight into quality of SRs published prior to the onset of the pandemic in December 2019. Where articles were found in each of the two datasets derived from KSR Evidence to be meta-analyses only, these articles were excluded from this comparison and not considered further.

Each dataset represented an export of data that was related to the SR. It included multiple data including name, year and location of publication, details of populations, interventions/exposures, comparators, outcomes, results, and relevant study designs as well as full ROBIS appraisal results. Briefly, where a SR is identified for inclusion in KSR Evidence, a full ROBIS appraisal is conducted by a reviewer and then independently checked by a second reviewer. The review is then audited by a third reviewer to provide a further level of scrutiny that the initial appraisal and independent check are appropriate.

### Analysis

We counted the frequency of ‘high’, ‘unclear’, and ‘low’ overall ROB gradings that were reported for all COVID-19-related, COVID-19-unrelated, and 2018 SRs. Where overall gradings were deemed to be of a ‘high’ or ‘unclear’ RoB, we adopted the conservative position that if optimal methodology is not clearly reported, then it cannot be relied upon to have been carried out, and therefore, a potential bias must be considered. For this reason, all the ‘high’ and ‘unclear’ categories of overall RoB were collapsed into a single category of ‘not low RoB’. Risks of an overall ‘low RoB’ were then calculated in each group, and the risk ratio (RR) of a low RoB was calculated for (1) COVID-19-related SRs versus COVID-19-unrelated SRs and (2) for all pandemic SRs (COVID-19 related and unrelated combined) versus pre-pandemic 2018 SRs. All 95% confidence intervals (CIs) were fitted around the RR.

As a post hoc consideration, we also counted the review types that were reported in each group to explore whether pandemic-related demands had changed research emphasis when compared to before the pandemic. Percentage distributions of each reported review type were tabulated and compared. We considered a percentage difference of more than 5% to represent a difference to be noteworthy. We emphasise that no formal statistical analysis was conducted when examining review types, and so any differences of 5% represent only what may be interesting observations.

## Results

A total of 2045 SRs were obtained that were published during the pandemic, while 1580 SRs were obtained that were published pre-pandemic. To address questions 1 and 3, we examined 318 SR’s that were related to COVID-19 published during the pandemic, compared to 1727 that were unrelated. To address questions 2 and 4, we compared all 2045 SRs published during the pandemic with all 1580 that were published pre-pandemic.

### Question 1: How does overall quality of COVID-19-related SRs published during the pandemic compare to those of COVID-19-unrelated SRs published during the pandemic?

Table 1 summarises the RoB ratings for the 318 COVID-19-related SRs and the 1727 COVID-19-unrelated SRs. The RR of a low RoB was calculated for COVID-19-related SRs versus COVID-19-unrelated SRs (Table 2). The RR of a low RoB for COVID-19-related SRs versus COVID-19-unrelated SRs was 0.94 (95% *CI*: 0.66 to 1.34). Although the point estimate indicates a slightly lower probability of a low RoB in COVID-19-related SRs compared to COVID-19-unrelated SRs within the studied sample, the confidence intervals suggest a result that is consistent with the sample being drawn from a population with no difference in RoB across the two groups.

**Table 1:** RoB ratings across COVID-19-related SRs and COVID-19-unrelated SRs

ROBIS RoB rating	COVID-19-related SRs	COVID-19-unrelated SRs
Low	32 (10.06%)	185 (10.71%)
Unclear	16 (5.03%)	109 (6.31%)
High	270 (84.9%)	1433 (82.97%)
Total	318	1727
Risk of a low RoB	32/318 = 0.101	185/1727 = 0.107
Risk ratio for COVID-19-related SRs versus COVID-19-unrelated SRs	0.101/0.107 = 0.94

**Table 2:** RoB ratings across all SRs during the pandemic and all SRs pre-pandemic

ROBIS RoB rating	All SRs during pandemic	All SRs pre-pandemic
Low	217 (10.61%)	565 (35.75%)
Unclear	125 (6.11%)	105 (6.64%)
High	1703 (83.27%)	910 (57.59%)
Total	2045	1580
Risk of a low RoB	217/2045 = 0.106	565/1580 = 0.358
Risk ratio for pandemic SRs versus pre-pandemic SRs	0.106/0.358 = 0.30	

### Question 2: How does overall quality of SRs published during the pandemic compare to those SRs published prior to the pandemic?

Table 2 summarises the RoB ratings for the 2045 pandemic SRs and the 1580 pre-pandemic SRs. The RR of a low RoB was calculated for pandemic SRs versus pre-pandemic SRs (Table 2). The RR of a low RoB for pandemic SRs versus pre-pandemic SRs was 0.30 (95% *CI*: 0.26 to 0.34). This result suggests that SRs were more likely to be low risk pre-pandemic, compared to during the pandemic.

Table 3 summarises the frequency of review types found in the two datasets. Results are presented for the COVID-19 sample, the COVID-19-unrelated sample, these samples combined (pandemic), and the pre-pandemic sample. Review types are categorised by intervention, aetiological, epidemiological, prognostic/predictive, diagnostic, unclear, or other. These categorisations were made during the appraisal process by the reviewers at the time.

**Table 3:** Review type for all reviews included within this comparison that are present on KSR Evidence

Review type	COVID-19 related (pandemic)	COVID-19 unrelated (pandemic)	COVID-19 related and COVID-19 unrelated combined (pandemic)	Pre-pandemic
**Total number**	**318**	**1727**	**2045**	**1580**
Intervention	39.43%	69.8%	65.12%	72.47%
Aetiological	15.46%	7.45%	8.76%	6.46%
Epidemiological	11.99%	3.18%	4.55%	3.61%
Prognostic/predictive	12.93%	7.30%	8.17%	7.09%
Diagnostic	7.89%	2.61%	3.42%	4.18%
Unclear/other	12.03%	9.66%	9.98%	6.19%

### Question 3: How does the review type distribution of COVID-19-related SRs compare to those of COVID-19-unrelated SRs published during the pandemic?

The number of COVID-19-related reviews was considerably lower (318) when compared to the COVID-19-unrelated reviews (1727). Noticeable percentage distribution differences existed in every category of review when comparing between COVID-19 and COVID-19-unrelated reviews. SRs of interventions were the most common review type across each of the groups, although the percentage distributions were different with less intervention reviews being published related to COVID-19 (39.43%) when compared with COVID-19 unrelated (69.8%). Aetiological (COVID-19: 15.46% vs. 7.45%) and epidemiological (COVID-19: 11.99% vs. 3.18%) review types were the next most common review types in the COVID-19 groups with more than twice as many of these review types in the COVID-19 groups, compared to the COVID-19-unrelated group. There were greater prognostic/predictive reviews published pertaining to COVID-19 (12.93%) than to COVID-19 unrelated (7.30%) and of diagnostic reviews (COVID-19: 7.89% vs. 2.61%). Reviews marked as ‘unclear/other’ were generally similar (12.03% vs. 9.03%) in both groups.

### Question 4: How does the review type distribution of SRs published during the pandemic compare to those SRs published prior to the pandemic?

There was a greater absolute number of SRs in the pandemic sample than prior to the pandemic (2045 vs. 1580). However, when considering the mean number by month, it is apparent that there were more articles published monthly in the pre-pandemic sample. Our pandemic sample consisted of 30.5 months of records and a total number of 2045 records meaning a crude average of 67.04 records for each month. The pre-pandemic sample consisted of 1580 records over a 12-month period of 2018 with a crude average of 131.66 records per month. SRs of interventions were the most common review type across both groups, although the percentage distributions were different with more reviews of interventions being present before the pandemic (72.47% vs. 65.12%). There were no other marked differences (> 5%) observed between the pre- and pandemic review types. A total of 8.76% of all pandemic reviews were related to aetiological research compared to 6.46% of pre-pandemic research, while reviews categorised as ‘epidemiological’ represented 4.55% of all pandemic reviews compared to 3.55% of all pre-pandemic literature. Prognostic/predictive distributions (8.17% vs. 7.09%) as well as diagnostic distributions (3.42% vs. 4.18%) were broadly similar with no marked differences. Reviews marked as unclear/other constituted 9.98% of all pandemic reviews, with 6.19% of all pre-pandemic reviews categorised as such.

## Discussion

We conducted an exploratory analysis on the quality of SRs that were published related to COVID-19 to determine if there was any difference in overall quality compared to those that were unrelated to COVID-19. Our secondary interest was also to determine if any differences in overall quality existed between those SRs that were published during the pandemic compared to those that were published prior to the pandemic.

### Summary of main findings

We examined the overall quality of COVID-19-related SRs published during the pandemic compared to those that were unrelated to COVID-19. Surprisingly, no marked difference in overall quality was found. The RR of a low RoB for COVID-19-related SRs versus COVID-19-unrelated SRs was 0.94 (95% *CI*: 0.66 to 1.34). The analysis demonstrated that most COVID-19 SRs were of a low overall quality with only 10% rated with a low RoB. However, most COVID-19-unrelated SRs were also of a low overall quality with 11% rated as low RoB. This shows that SR quality was equally low for both COVID-19-related and COVID-19-unrelated papers during the pandemic. The obvious question would therefore be to ask ‘whether *all* SRs in general conducted during the pandemic are of lower quality than those published pre-pandemic?’.

Quality differences were more pronounced with 11% of pandemic reviews being of low RoB compared to 36% of pre-pandemic reviews, leading to the RR of a low RoB for pandemic reviews versus pre-pandemic reviews of 0.30 (95% *CI*: 0.26 to 0.34). The pandemic introduced a range of restrictions and difficulties across all aspects of life, and it is feasible that this influenced quality differences. While concerns have been raised in the literature regarding the quality of COVID-19 primary research [13, 24] and SRs [15–18], it is perhaps reasonable to suggest that all SR research conducted and produced during the restrictions of the pandemic may have experienced quality and methodological issues. Certainly, our data would suggest clear differences between the pandemic and pre-pandemic periods in terms of the rates of overall quality. The pandemic sample consisted of a crude average of 67.04 records for each month, whereas the pre-pandemic sample consisted of a crude average of 131.66 records per month. Two-hundred seventeen SR’s were rated as low RoB during the 30.5 months of the pandemic sample, meaning a crude average of 7.11 low RoB publications per month. The pre-pandemic sample consisted of 565 SR’s rated as low RoB over a 12-month period, meaning that a crude average of 47 low RoB SR’s was published per month in 2018.

We explored the distribution of review types across the pandemic sample to understand if COVID-19 had generated a pressure for particular SR research. With the consideration of a 5% percentage difference being of note, there were some clear differences. There were more intervention SRs in the COVID-19-unrelated group compared to the COVID-19 groups. Intervention SRs remained the largest proportion of SRs in each group; however, the distribution was markedly different with 39.43% of COVID-19 SRs being focused on interventions compared to 69.8% of COVID-19-unrelated SRs. This finding broadly reflects the work of Dang and colleagues [15] who in their analysis of COVID-19 SRs reported that 39.58% were interventional but did not compare against COVID-19-unrelated SRs. A possible explanation for this can be found in our sample timeframe from March 2020 to September 2022. This included the earlier stages of the pandemic where interventions were still in an early phase and no consistent, established evidence-based regimen had been conclusively confirmed. In fact, we consider that COVID-19 research in the earlier stages of the pandemic was more likely to be focused on issues of transmission, susceptibility, risks, and population dynamics of infection (i.e. epidemiological/aetiological/prognostic factors).

It is apparent that publications related to COVID-19 are increased in the areas of epidemiology and aetiology compared to COVID-19-unrelated reviews (Table 1). This is supported by evidence from previous research [17]. Baumeister and colleagues explored the quality of 439 SRs related to COVID-19 up to July 2020 and appraised by the AMSTAR-2 tool. While they used different terms and criteria for the categorisation of the included SRs, they reported that around 70% were concerned with prognostic and epidemiological parameters indicating that most reviews in the earlier stages of the pandemic were indeed related to matters of epidemiology, aetiology, transmission, risk factors, etc. As the pandemic continued, research will likely have shifted towards treatment and interventions; however, as our sample still included the earlier stages of the pandemic, we see COVID-19 intervention reviews at a considerably lower level than that of COVID-19-unrelated reviews. We would consider it reasonable to suggest that a review of a later dataset may potentially see reviews of interventions increase towards that of the COVID-19-unrelated levels.

Finally, we explored the distribution of SR types between the pandemic and pre-pandemic samples. However, we found that the distribution of SR types was generally similar for all types, except for interventional reviews. A total of 72.47% of pre-pandemic reviews were interventional, while 65.12% of pandemic reviews were categorised as such.

### Comparison to previous research

Our analysis further confirms the findings of previous research where COVID-19 SRs have been demonstrated to be of poor quality. Baumeister and colleagues [17] in their analysis reported that when COVID-19 SRs were appraised using the AMSTAR-2 tool, only 151/439 were rated as high quality (34.39%), while 51.25% (251/439) were rated as either ‘very low’ or ‘low’ quality. Chen and colleagues [18] conducted a cross-sectional analysis on all SRs and meta-analyses that were published up to May 2020 and included articles of varying designs. They utilised different appraisal tools depending on study design and reported that of the 47 articles they included, only 6.4% were of a high quality, with the remainder being of low or critically low quality. Dang et al. [15] reported the low quality of COVID-19 SRs. In their analysis of 48 SRs about COVID-19, they reported that only 10.42% had a low RoB when appraised by the ROBIS tool. Li and colleagues [16] explored the quality and evidence mapping of COVID-19 SRs and included 243 SRs in their analysis. They reported that 87.6% of SRs were of low or critically low quality when assessed by AMSTAR-2 with 12.3% of moderate quality.

Our data confirm and support these previous findings with our results demonstrating only 10.06% of SRs had a low RoB. Chen and Dang et al., however, only examined SRs in the earlier stages of the pandemic with a final search end date of May 2020, while Li and Baumeister had final search dates of June and July 2020 respectively — which represented arguably the most acute stage of the pandemic in terms of preliminary evidence with considerable unknown variables regarding the transmission and impact of COVID-19. Research efforts in this period had increased dramatically, at an increased pace, and with an urgency unparalleled in living memory. The pressing need to understand the mechanisms of transmission, patient characteristics, disease severity and trajectory, and the population dynamics surrounding this would be a plausible explanation. Furthermore, neither of these previous studies compared against COVID-19-unrelated research.

Our analysis, however, goes further to 14th September 2022, and yet there was no time influenced improvement in overall quality up to that point. Furthermore, we compared the quality against COVID-19-unrelated SRs and found that there was no difference in quality with both sets of SRs lacking in methodological rigour. This adds a different perspective to the concerns regarding the quality of COVID-19 SRs. It can indeed be argued that they are of low quality, when considering overall appraisal score distribution, but they are not *lower quality* than other SR research published during the same period. However, we note that the reasons for similar quality scores may be different with the possibility that the origins of quality impairment may be derived from different ROBIS domains or signalling items.

The finding that marked differences in overall quality between the pandemic and pre-pandemic timeframes may suggest that external pandemic-related pressures affected all SR research negatively to some extent. However, perhaps a further concern that can be highlighted when considering that the pre-pandemic sample observed a higher rate of better-quality work, with 36% having a ROBIS appraised low RoB, is that 64% of SRs did not have a low RoB. SRs are at the top of the evidence pyramid, considered to be of the highest calibre and are expected to identify and examine all the available evidence, conducted and reported with such rigour that they can be relied upon for decision-making of significant impact. Despite this, our data demonstrate and reinforce that in fact the general quality of SRs would appear to be poor and often unreliable, and this is a concern in evidence-based medicine or decision-making more widely. SRs have been shown in previous analyses to be of lower quality and unreliable for informing decision-making [25], while SRs in areas as diverse as human immunodeficiency virus (HIV) and digital health have also been found to be poor [26–28]. It is feasible that rigorous SR research is being inadequately reported. Likewise, it is also feasible that insufficiently designed and executed research is indeed reported adequately so that it is accurately appraised as being lower quality. We did not explore this. Ultimately, any research, irrespective of how well designed and executed it may be, is limited by its ability to be disseminated with confidence clarity and relevance and so is ‘only as good as its reporting’.

### Strengths and limitations

This exploratory comparison has provided only a rudimentary and basic analysis of SRs that were held within one database (KSR Evidence), were already appraised, and were available to the authors. All SRs that were included in this analysis had been marked as ‘priority’ screens, and so it is possible that other relevant SRs have not been included because not initially marked as priority. We have also assumed that those SRs tagged in the database as COVID-19 SRs or not tagged as COVID-19 SRs are as described, and we have not verified or explored any of the articles. Of those that were included, we are conscious of the limited sample and final inclusion date of 14th September 2022, meaning SRs from the remainder of 2022 and 2023 are not included. We are also aware of our pre-pandemic sample being exclusively from 1 year (2018). We recognise that this exploratory analysis may include SRs in the COVID-19-unrelated category that were published during the pandemic but conducted written and/or submitted for publication prior to the pandemic. We do not consider that this will constitute any meaningful number or hold any meaningful impact, and we remind readers that this exploration is examining SRs by when published, not when conducted. All appraisals were conducted using the ROBIS tool which suggests SRs have high, low, or unclear RoB. Any missing information can therefore render a particular item, domain, and thus the overall score to be conservatively rated at high RoB, and we have not examined the differences in individual signalling questions between the groups to determine where potential quality impairments in each domain may have occurred. Furthermore, we did not apply any selection criteria to the SRs available in the database, other than to remove all entries which were listed as ‘meta-analysis only’. Therefore, it is feasible that some SRs included in this analysis were not actually SRs and were ‘other reviews’ which have described themselves, or been incorrectly defined, as systematic. We also did not distinguish between the type of SR design, nature of included studies, or scientific or healthcare discipline. Thus, there may be wide heterogeneity within and between each sample with regard to these topics.

Despite these limitations, we consider that our exploration does possess strengths. Firstly, the KSR Evidence review process relies on multi-level appraisal and quality control, and so we are confident that all articles are as described. Secondly, we have compared COVID-19 SRs against COVID-19-unrelated SRs, as well as SRs published during the pandemic against those published prior to the pandemic. Finally, we emphasise to readers that this exploratory analysis is limited to being exactly as follows: an introductory exploration designed to provide an insight into an issue of overall SR quality against the backdrop of the COVID-19 pandemic. We are aware that further analysis should build upon this, and we intend to conduct further investigations into quality across individual items within each domain, as well as to gain insight into the relationship between review type and quality. Furthermore, we intend to extract additional data from KSR Evidence to explore whether quality has remained low since the end of the pandemic or has begun to climb more towards pre-pandemic levels. This additional research will form the basis for an update to this introductory exploration which we intend to publish in the future.

## Conclusions

These results suggest no statistically significant difference in overall quality between COVID-19-related SRs and SRs unrelated to COVID-19 when appraised by ROBIS. These results do however reinforce previous findings of poor quality COVID-19 SR evidence albeit alongside equally poor quality COVID-19-unrelated SRs. Differences in SR quality between pandemic and pre-pandemic timeframes were observed suggesting a possible impact of pandemic-related pressures on quality of all areas of research, rather than just on COVID-19 research. To the best of our knowledge, this is the first exploratory analysis to compare COVID-19 SR quality with of SRs unrelated to COVID-19, as well as pandemic versus pre-pandemic quality, including published articles up to September 2022, and utilising the ROBIS tool. Our findings also emphasise the general overall low quality of SRs, which may reflect a potentially more serious issue in the evidence synthesis process given that poorly conducted and/or reported reviews may be informing important decisions. Researchers and journal editors should ensure that appropriate standards are met in conducting, reporting, and publishing SRs, and previously published SR evidence should potentially be revisited. It should also be underlined that those who use SRs to inform policy, guidelines, clinical protocols, and other important decision-making should exercise caution, conduct the appropriate appraisals, and should remain critical.

## Data Availability

The datasets used and/or analysed during the current study are available from the corresponding author on reasonable request.

## Abbreviations

AMSTAR 2: A Measurement Tool to Assess systematic Reviews
CI: Confidence interval
KSR: Kleijnen Systematic Reviews
PRISMA: Preferred Reporting Items for Systematic Reviews and Meta-Analysis
RoB: Risk of bias
RR: Risk ratio
SR: Systematic review
